# Impact of new health care reform on enabling environment for children’s health in China: An interrupted time-series study

**DOI:** 10.7189/jogh.12.11002

**Published:** 2022-03-19

**Authors:** Huihui Huangfu, Zhifan Zhang, Qinwen Yu, Qingyu Zhou, Peiwu Shi, Qunhong Shen, Zhaoyang Zhang, Zheng Chen, Chuan Pu, Lingzhong Xu, Zhi Hu, Anning Ma, Zhaohui Gong, Tianqiang Xu, Panshi Wang, Hua Wang, Chao Hao, Chengyue Li, Mo Hao

**Affiliations:** 1 Research Institute of Health Development Strategies, Fudan University, Shanghai, China; 2 Collaborative Innovation Center of Social Risks Governance in Health, Fudan University, Shanghai, China; 3 Zhejiang Academy of Medical Sciences, Hangzhou, Zhejiang, China; 4 School of Public Policy and Management, Tsinghua University, Beijing, China; 5 Project Supervision Center of National Health Commission of the People’s Republic of China, Beijing, China; 6 Department of Grassroots Public Health Management Group, Public Health Management Branch of Chinese Preventive Medicine Association, Shanghai, China; 7 School of Public Health and Management, Chongqing Medical University, Chongqing, China; 8 School of Public Health, Shandong University, Jinan, Shandong, China; 9 School of Health Service Management, Anhui Medical University, Hefei, Anhui, China; 10 School of Management, Weifang Medical University, Weifang, Shandong, China; 11 Committee on Medicine and Health of Central Committee of China ZHI GONG PARTY, Beijing, China; 12 Institute of Inspection and Supervision, Shanghai Municipal Health Commission, Shanghai, China; 13 Shanghai Municipal Health Commission, Shanghai, China; 14 Jiangsu Preventive Medicine Association, Nanjing, Jiangsu, China; 15 Changzhou Center for Disease Control and Prevention, Changzhou, Jiangsu, China

## Abstract

**Background:**

Creating an enabling environment (EE) can help foster the development and health of children. The Chinese government implemented a new health care reform (NHR) in 2009 in a move to promote an EE for health. The purpose of this study was to evaluate the impact of the NHR on EE for children’s health.

**Methods:**

An interrupted time-series analysis was used to evaluate the changes in the EE before and after 2009 in China. This study analysed the EE through five quantitative indicators, including policy element coverage rate (PECR), service meeting with children’s needs rate (SMCNR), multisector participation rate (MPR), and accountability mechanism clarity rate (AMCR), based on the content analysis of available public policy documents (updated as of 2019) from 31 provinces in mainland China, and the number of health care personnel of maternity and child care centres per 10 000 population (HP per 10 000 population), based on the 2002–2019 China Health Statistical Yearbook and China Statistical Yearbook.

**Results:**

The average values of PECR, SMCNR, and MPR increased rapidly to 90.96%, 82.46%, and 81.31%, respectively, in 2019, representing a higher value compared to the AMCR (7.38%). The NHR promoted the EE, in which HP per 10 000 population showed the fastest increase (β_1_ = 0.03, *P* < 0.01; β_3_ = 0.10, *P* < 0.01), followed by SMCNR (β_1_ = 0.94, *P* < 0.01; β_3_ = 1.83, *P* < 0.01), AMCR (β_1_ = 0.13, *P* < 0.01; β_3_ = 0.24, *P* = 0.14), MPR (β_1_ = 1.35, *P* < 0.01; β_3_ = 2.47, *P* < 0.01) and PECR (β_1_ = 1.43, *P* < 0.01; β_3_ = 1.47, *P* < 0.01).

**Conclusions:**

The NHR has a positive impact on the EE, especially on the human resources and service provision for children. Efforts should be intensified to improve the clarity of the accountability mechanism of the health-related sectors.

Children’s health and future are intimately linked to the health of our planet [1]. In 2020, nearly 40% of children in China faced risks such as early stunting [2]. If these risks are not effectively controlled, by 2040, nearly half of China’s workforce will be compromised. This not only hinders the country’s development, but also widens inequality, exposing China to the risk of instability [3].

Creating an enabling environment (EE) can promote the development and health of children [4]. In 2010, the United Nations launched the *Every Woman Every Child* movement to mobilise governments and sectors to jointly create an EE for children [5]. According to the *Global Strategy for Women’s, Children’s and Adolescents’ Health (2016-2030)*, an EE within the health system and other sectors would strengthen the investments in children’s health, including health policies, resource allocation, health service provision, multisector collaboration, etc [6].

China has progressed in the direction. At present, China has developed a relatively complete policy and legal system in the field of women and children [7]. The new health care reform (NHR) was launched in 2009 by the Chinese government, and aimed to improve the network of public health services for maternal and children. Meanwhile, the reform also focused on promoting equitable access to essential health services, including health records, health education, health management, etc. For example, the government established a child health manual for infants under 3 years old, carried out neonatal visits and child health system management, and vaccinated school-aged children under a national immunisation programme [8,9]. By 2019, the under-five mortality rate in China dropped from 17.2‰ (in 2009) to 7.8‰ [10]. This indicated that after the NHR, the EE in China has realised the importance of children’s rights to health and well-being [10].

Research on the EE mainly focused on the current situation of health problems experienced by children at different stages of life, such as adolescent sexual and reproductive health [4], infant and young child nutrition [11-13], breastfeeding [14,15], and childhood stunting [16]. Some studies have analysed the health-related changes in the EE before and after implementing the policies. Zhao et al. found that the essential public health service project had a positive impact on neonatal health services and in reducing neonatal mortality [17]. Sia et al. and Druetz et al. considered that the free health care policy characterised by free consultation, care, medication and laboratory and radiological examinations (including ultrasounds) implemented in 2016 in Burkina Faso had increased the usage of health services for children aged under 5 years [18,19]. The input of resources has increased, and the equity of resource allocation has also improved. For example, Yang et al. found that the number of health care personnel in township hospital centres in China increased after the NHR [20]. Meanwhile, Fu et al. and Zhao et al. reported that health resource allocation has improved [21-23]. Health sectors began to show the trend of multisector collaborative governance after the reform [24]. To the best of our knowledge, many studies have focused on the impact of policy implementation on a single aspect of the EE, such as service provision and utilisation, resource allocation, and multisector collaboration. However, there is little comprehensively analysed evidence on how the NHR affected the EE. Thus, through this study, we attempted to fill this gap and assess the impact of NHR on children’s health from the perspective of EE.

## METHODS

### Study design and setting

We used an interrupted time-series (ITS) analysis to evaluate the changes brought by the NHR on the EE in China. In 2009, the State Council released *the Opinions on Deepening the Reform of the Medical and Health Care System*, in which the government proposed to improve the network of public health service for maternal and children and focused on promoting equitable access to essential health services [8]. We considered 31 provinces in mainland China as the study setting and analysed the changes in the EE before (2002–2008) and after (2009–2019) the NHR was implemented. We assumed that any time-varying unmeasured confounder changed relatively slowly [25].

### Measurements

According to *the Global Strategy for Women’s, Children’s and Adolescents’ Health (2016-2030)*, EE for children’s health includes policies for universal health coverage, mainstreaming emergency preparedness, human rights, equity and gender-based approaches in programming, sufficient and sustainable financing, human resources, health facility infrastructure, good-quality care provision, community engagement and accountability at all levels, and multisector promotion strategies, including finance and social protection, education, gender, protection registration, law and justice, water and sanitation, agriculture and nutrition, environment and energy, labour and trade, infrastructure, information and communication technologies, and transport [6]. Therefore, we designed five indicators to reflect the situation of the EE (**Table 1**). The larger the indicator value, the better is the EE for children’s health.

**Table 1:** Definition of five indicators of enabling environment.

Indicator name	Indicator definition
Policy element coverage rate (PECR) (%)	The proportion of the number of policy elements covered in children’s health policy document collection to the 25 required elements [26,27]
Number of health care personnel of maternity and child care centres per 10000 population (HP per 10000 population) (person)	The number of health care personnel in maternal and child health care centres per 10000 population
Service meeting with children’s needs rate (SMCNR) (%)	The average of services coverage rate and the assessable health service coverage rate among the eight types of services
Multisector participation rate (MPR) (%)	The proportion of the number of departments to the 15 departments that should be included in children’s health
Accountability mechanism clarity rate (AMCR) (%)	The proportion of the number of departments with clearly defined monitoring agencies and accountabilities to the 15 departments that should be included in children’s health

Policies for universal health coverage, mainstreaming emergency preparedness, and human rights, equity, and gender-based approaches in programming are the political guarantees for health [28]. This requires a good policy environment to provide guarantees regarding objectives, tasks and measures, organisation and resource allocation, department responsibilities and accountability, department assessment and incentive, etc [26]. Therefore, we used the policy element coverage rate (PECR) to reflect the three elements.

Sufficient and sustainable financing, human resources, and health facility infrastructure represent the resource guarantees for health [29]. Human resources are the primary and critical resources, while financing and health facility infrastructure must be manipulated and dominated by human resources [30]. Therefore, we used the number of health care personnel of maternity and child care centres per 10 000 population (HP per 10 000 population) to reflect the resource allocation.

Good-quality care provision is the service guarantee for health [28]. This requires health services delivery that meets the physical and psychological needs of children. Therefore, we used the service meeting with the children’s needs rate (SMCNR) to reflect the provision of good-quality care. According to the guidelines and agendas for children’s health issued by the World Health Organization (WHO), China, the United States, and the United Kingdom [6,31-33], as well as expert consultation, at least eight types of services should be provided, including birth defect screening and management, exclusive breastfeeding guidance, specialised case management for high-risk children, nutrition guidance for infants, infant growth and development monitoring, early childhood development, child growth and development monitoring, and mental behavioural development evaluation and guidance (Table S1 in the **Online Supplementary Document**).

Community engagement and multisector promotion measures are the embodiment of multisector participation and coordination, and provide the structural foundation for promoting children’s health [34]. Therefore, we used the multisector participation rate (MPR) to reflect sector participation. There should be at least 15 stakeholders involved in children’s health according to WHO and China [3,6,35-37], including the government, health commission, public health agencies, hospitals, primary health care institutions, policy security department, finance bureau, human resources and social security bureau, health care security administration, education commission, civil affairs bureau, agriculture and rural affairs bureau, construction department, and water resources department, among others.

Accountability at all levels is crucial to ensure that sectors meet their commitments and put them into practice [6]. This requires the sectors to have clear responsibility and accountability. Therefore, we used the accountability mechanism clarity rate (AMCR) to reflect accountability at all levels.

### Data collection

We obtained the PECR, SMCNR, MPR, and AMCR data from the content analysis of policy documents collected from official websites of the legislature and legal databases of 31 provinces in mainland China (updated as of 2019). The types of policy documents included laws, regulations, strategies, norms, rules, plans, budgets, guidelines, and standards [38]. Policy documents which were duplicate, or without administrative validity or a specific publication date, were excluded [38]. Finally, a total of 1393 documents were collected in our study (Figure S1 in the **Online Supplementary Document**).

We extracted the information needed for the four indicators from policy documents and provided detailed information coding in Table S2 in the **Online Supplementary Document**. Taking MPR as an example, we determined whether 15 categories of departments were referred to in the documents. If it mentioned that health services were organised and implemented by the hospitals, they were considered as a stakeholder in children’s health. Thus, we coded the hospitals as 1. The number of departments mentioned divided by 15 (number of departments) constituted the MPR value. The test-retest reliability method with the intraclass correlation coefficient (ICC) was used to analyse the credibility of the data collection. After retesting by two experienced researchers, the ICC value was higher than 0.75, indicating a highly credible data collection.

We obtained the number of health care personnel of maternity and child care centres and the total population at the year-end data from the 2002–2019 China Health Statistical Yearbook and China Statistical Yearbook.

### Statistical analysis

ITS can be considered a quasi-experimental research design with a high degree of internal validity to assess the longitudinal effects of interventions [39]. Thus, we used the ITS analysis to assess the level and trend changes in EE before and after the NHR, and performed the Cumby-Huizinga test for autocorrelation [40]. We regarded 2009 as the intervention time point. The specific formula applied was as follows:

Y_t_ = β_0_+ β_1_ × time + β_2_ × intervention + β_3_ × time_after_intervention + ε_it_

Y_t_ was the outcome variable measured at time point t. β_0_ represented the level of the outcome at the start time. β_1_ represented the trend of outcome preintervention. β_2_ represented a change in the level of outcome postintervention. β_3_ represented a change in the trend of outcome postintervention. ε_it_ was an estimate of random error at time t.

Data were analysed using Excel 2019 (Microsoft, Redmond, WA, USA) and Stata 14.0 (Stata Corp., College Station, TX, USA). Given the small sample size, the level of statistical significance was set at *P* < 0.1.

## RESULTS

### Description analysis of EE

**Table 2** shows that the average values of PECR, HP per 10 000 population, SMCNR, MPR, and AMCR in 2002 were 42.55%, 1.22 person, 41.02%, 34.23%, and 1.19%, respectively. The average values of PECR, SMCNR, and MPR in 2019 were over 80%, 90.96%, 82.46%, and 81.31%, respectively, while the AMCR value was 7.38%. HP per 10 000 population in 2019 was 2.66 person. The EE for children’s health has improved since 2002, with the largest increment in AMCR (518.06%) and the smallest in SMCNR (101.05%).

**Table 2:** Description statistics of enabling environment from 2002 to 2019

Indicators	2002	2019	Increment since 2002
PECR (%)	42.55	90.96	113.78
HP per 10000 population (person)	1.22	2.66	118.30
SMCNR (%)	41.02	82.46	101.05
MPR (%)	34.23	81.31	137.57
AMCR (%)	1.19	7.38	518.06

PECR – policy element coverage rate, HP – health care personnel, SMCNR – service meeting with children’s needs rate, MPR – multisector participation rate, AMCR – accountability mechanism clarity rate

### Analysis of the impact of reform on EE

**Figure 1:**
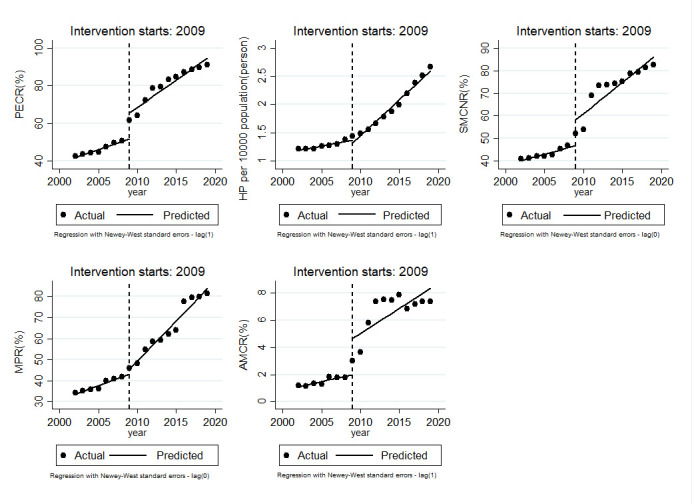
Level and trend changes of five indicators pre- and post-intervention. PECR – policy element coverage rate, HP – health care personnel, SMCNR – service meeting with children needs rate, MPR – multisector participation rate, AMCR – accountability mechanism clarity rate.

**Table 3** and **Figure 1** show the ITS analysis results of the impact of reform on the EE. The trend of all indicators slowly increased before the NHR in PECR (1.43, 95% confidence interval (CI) = 1.14 to 1.72, *P* < 0.01), HP per 10 000 population (0.03, 95% CI = 0.01 to 0.04, *P* < 0.01), SMCNR (0.94, 95% CI = 0.61 to 1.27, *P* < 0.01), MPR (1.35, 95% CI = 1.15 to 1.56, *P* < 0.01), and AMCR (0.13, 95% CI = 0.09 to 0.16, *P* < 0.01). After the reform, there was an immediate increase in PECR (13.79, 95% CI = 7.72 to 19.85, *P* < 0.01), SMCNR (11.42, 95% CI = 3.18 to 19.66, *P* < 0.05), MPR (2.43, 95% CI = 0.17 to 4.68, *P* < 0.05), and AMCR (2.66, 95% CI = 0.44 to 4.89, *P* < 0.05). The trend of all the rates showed a rapid increase in PECR (1.47, 95% CI = 0.57 to 2.37, *P* < 0.01), HP per 10 000 population (0.10, 95% CI = 0.08 to 0.13, *P* < 0.01), SMCNR (1.83, 95% CI = 0.66 to 2.99, *P* < 0.01), and MPR (2.47, 95% CI = 1.95 to 2.98, *P* < 0.01). The two fastest increasing indicators were HP per 10 000 population (3.33, 0.10/0.03) and SMCNR (1.95, 1.83/0.94).

**Table 3:** Estimated level and trend changes of enabling environment pre- and post-intervention

Indicators	Baseline level (β_0_)	Baseline trend (β_1_)	Post policy level change (β_2_)	Post policy trend change (β_3_)
PECR (%)	41.84† (40.63-43.05)	1.43† (1.14-1.72)	13.79† (7.72-19.85)	1.47† (0.57-2.37)
HP per 10000 population(person)	1.19† (1.15-1.23)	0.03† (0.01-0.04)	-0.05 (-0.16-0.06)	0.10† (0.08-0.13)
SMCNR (%)	40.27† (39.27-41.26)	0.94† (0.61-1.27)	11.42* (3.18-19.66)	1.83† (0.66-2.99)
MPR (%)	33.65† (32.68-34.62)	1.35† (1.15-1.56)	2.43* (0.17-4.68)	2.47† (1.95-2.98)
AMCR (%)	1.10† (0.98-1.23)	0.13† (0.09-0.16)	2.66* (0.44-4.89)	0.24 (-0.08-0.56)

PECR – policy element coverage rate, HP – health care personnel, SMCNR – service meeting with children’s needs rate, MPR – multisector participation rate, AMCR – accounttability mechanism clarity rate

*P < 0.05-

†*P* < 0.01.

## DISCUSSION

To our knowledge, this study is the first to use public policy documents, instead of the survey data, to quantitatively analyse the changes in the EE before and after the reform in China.

The NHR played a significant role in promoting an EE for the development and health of children, which is consistent with the research conclusions of Jin et al. [7], indicating that the government valued children’s health. First, the Chinese government incorporated children’s health into national economic and social development planning as a priority area [36,41,42]. The health sector also formulated a series of supporting regulations and policies [31,43], ensuring that children’s health services can be effectively provided [7]. Second, the government promoted children’s health into action [22]. China implemented major and essential public health service projects for children. Managing the supply, storage and distribution of vaccines, drugs, and nutritional packages has strengthened material guarantee for children’s health [7].

The results of the ITS showed that policy implementation has a positive impact on resource allocation and service provision. After the reform, resource allocation for children has improved significantly. This is consistent with the conclusions of Song et al [44]. The government has increased its efforts to improve the conditions of health personnel, including personnel training, personnel incentives, etc [8]. In 2011, the government put forward measures to increase the number of maternal and child care institutions, children’s hospitals and health personnel [41]. In 2016, the government adopted incentive measures for paediatric health personnel, including raising salaries and benefits, resulting in improved internal distribution mechanism by focusing on the quantity and quality of services and patient satisfaction. Meanwhile, the government also promoted the development of health personnel and prioritised the promotion and selection of highly qualified professionals [45]. As a result, the number of health care personnel in maternal and child care centres has continued to grow [46], and the overall quality and stability has improved [47,48].

This study also showed that the provision of good-quality care for children has increased significantly after the policy implementation, consistent with the research conclusions of Wang et al. [49]. Since 2009, China has gradually improved the network of public health service for maternal and children and have provided equitable access to essential health services [7]. For example, the government has implemented a nutrition programme for children living in poverty-stricken areas. They provided a pack of nutritional dietary supplements containing proteins, vitamins, and minerals on a daily basis for every child aged 6–24 months, and imparted education on child nutrition [42]. The neonatal disease screening program in poverty-stricken areas provided free screening and early diagnosis for 3.39 million neonatal children with phenylketonuria, congenital hypothyroidism, etc., from 2012 to 2015 [7,43]. These indicated that the NHR has placed greater emphasis on health services fundamental for children [50].

The results of the policy content analysis showed that the AMCR in China needed to be improved, which is consistent with the research conclusions of Geng et al. [51]. This may be due to several reasons. First, the responsibilities of the finance bureau and health care security administration were not clear [52]. For example, the Finance Bureau was responsible for using funds to support the infant care services industry. However, it did not specify the investment quota and timelines for the finance department, indicating the absence of implementation specifications. Second, the departmental accountability mechanism was not satisfactory. The government has evaluated the quality of services offered by the health sector, while the evaluation of other related sectors in most provinces is not yet clear [51]. For example, Education Commission, Human Resources and Social Security Bureau, Civil Affairs Bureau, etc., should jointly implement the nutrition programme [53]. However, the absence of the monitoring and accountability mechanisms for these sectors resulted in the awful implementation of the programme. Furthermore, the mismatch between the evaluation results and salaries has also failed to mobilise staff [54]. Therefore, the accountability mechanism should be established to restrain the behaviour of the departments involved. The results of the assessment should be engaged in staff performance appraisal, in order to mobilise the effective implementation of various measures for the goal of promoting children’s health.

This study has some policy implications. First, the establishment and improvement of EE for children cannot be separated from the government, which should play the leading role in policy, investment, etc. Second, resource allocation is prerequisite for progress. Resources are the basis to establish the EE for children. A sustainable resource supply will ensure the healthy development of children. Third, multisectoral cooperation is necessary to establish the EE. This requires not only the participation of health-related sectors but also the that of other sectors, such as the Finance Bureau, Education Commission, etc, to achieve the health for all children.

This study had several limitations. First, this study mainly focused on the national level. However, it did not analyse of the changes brought by the NHR to a specific region or province. Second, confounding by other policy interventions may bias ITS analyses [55]. For instance, in 2011, *Chinese Children’s Development Outline (2011-2020)* released by the State Council might promote the creation of an EE for children. The impact of this policy cannot be completely eliminated in the application of ITS. Therefore, this study may have overestimated the impact of NHR interventions. However, one of the strengths of ITS studies is that they can better avoid the effect by typical confounding factors, such as population status or economic development, etc., which remain fairly constant and are normally taken into account when modelling the underlying long-term trend [56]. Therefore, the typical confounding factors have little effect on our study. Third, this study focused on the impact of a macro-policy-enabling environment. In future studies, more attention could be paid to changes in the EE from the community or individual level.

## CONCLUSIONS

Our study quantitatively analysed the impact of the NHR policy on EE for children’s health using an ITS. The findings showed that the reform had a positive impact on the EE. However, the accountability mechanism of departments needs to be improved. In the future, the government should continuously play a leading role in coordinating and clarifying multisector responsibilities, strengthening departmental accountability mechanisms, and providing better services and environment to meet the goal of improving children’s health.

## Additional material


Online Supplementary Document
