# Supplementary material for: Impact of new health care reform on enabling environment for children’s health in China: An interrupted time-series study
Source: J Glob Health. 2022 Mar 19;12:11002. doi: 10.7189/jogh.12.11002 (PMC8932608; doi:10.7189/jogh.12.11002)
Supplement: Online Supplementary Document [file jogh-12-11002-s001.pdf]

Figure S1. Flowchart for selection of policy documents.

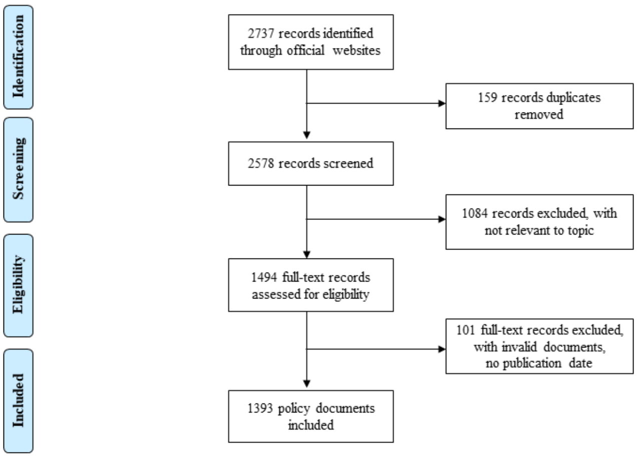

Table S1. Definition of eight types of children's health service

Service types	Main contents and measures
Birth defect screening and management	Popularize the prevention and treatment knowledge of birth defect, carry out targeted eugenics counselling services; carry out extensive prenatal screening, and popularize the application of appropriate prenatal screening techniques
Exclusive breastfeeding guidance	Provide breastfeeding information and enhance breastfeeding education and guidance
Specialised case management for high-risk children	Carry out special registration for high-risk children, such as low birth weight infants, premature infants and obesity, and strengthen management, including dietetic regulation, health education and follow-up visit, etc.
Nutrition guidance for infants	Provide corresponding nutritional suggestions for infants at different stages, including the choice of feeding methods, the time of complementary food and the supplement of nutrients
Infant growth and development monitoring	Use systematic methods, including tests, scales and physical examination to examine infants; carry out health education, health screening, regular or continuous close observation and treatment for infants' normal development

Early childhood development	Carry out scientific and comprehensive interventions for the characteristics of rapid physical and mental growth and development of infants and children aged 0–3, including physical, social, emotional, cognitive thinking, etc.
Child growth and development monitoring	Use systematic methods, including tests, scales, and physical examination to examine children; carry out health education, health screening, regular or continuous close observation, and treatment for children's normal development
Mental behavioural development evaluation and guidance	Provide psychological behaviour development counselling for children at different ages

Table S2. Information coding of indicators

Name	Description	Coding
Policy elements-A1	Record the content form	1-Long-term goal (over five years)<br>2-Short-term goal (under five years)<br>3-Put forward tasks and measure around th[illegible]<br>4-Policymaking<br>5-Service (intervention) content<br>6-Service (intervention) scope (region, population)<br>7-Service process<br>8-Operational norms<br>9-Technical standards<br>10-Institutional settings standards<br>11-Personnel allocation standards<br>12-Professional qualification standards<br>13-Funding sources<br>14-Funding standards<br>15-Funds guarantee measures<br>16-Material price standards<br>17-Material supply management norms<br>18-Information system construction standa[illegible]

		19-Information monitoring standards<br>20-Division of responsibility<br>21-Monitoring and control mode<br>22-Performance indexes and standards<br>23-Reward and punishment measures<br>24-Department coordination modes<br>25-Evaluation indicators and standards
Service types-A2	A2-1: Record service types:<br>•Birth defect screening and management<br>•Exclusive breastfeeding guidance<br>•Specialised case management for high-risk children<br>•Nutrition guidance for infants<br>•Infant growth and development monitoring<br>•Early childhood development<br>•Child growth and development monitoring<br>•Mental behavioural development evaluation and guidance	0-Not mentioned<br>1-Service mentioned in the policy docume
	A2-2: Record the assessable description of services	0-Not mentioned<br>1-Assessable service mentioned in the pol documents
Department participation-A3	Record department names:<br>•Provincial government<br>•Health commission<br>•Public health agencies<br>•Hospitals<br>•Primary health care institutions<br>•Finance bureau<br>•Human resources and social security bureau<br>•Policy security department<br>•Health care security administration	0-Not mentioned<br>1-Department participated in the policy documents

	•Education commission<br>•Civil affairs bureau<br>•Agriculture and rural affairs bureau<br>•Construction department<br>•Water resources department<br>•Non-government organisations	
Accountability mechanism-A4	Record the accountability mechanism of department<br>•Provincial government<br>•Health commission<br>•Public health agencies<br>•Hospitals<br>•Primary health care institutions<br>•Finance bureau<br>•Human resources and social security bureau<br>•Policy security department<br>•Health care security administration<br>•Education commission<br>•Civil affairs bureau<br>•Agriculture and rural affairs bureau<br>•Construction department<br>•Water resources department<br>•Non-government organisations	0-Not mentioned<br>1-Department accountability mechanism c[illegible] mentioned in the policy documents
